# A novel corporal dilation tool in penile implant surgery

**DOI:** 10.14744/nci.2022.68889

**Published:** 2022-04-20

**Authors:** Muhammet Ihsan Karaman, Orhan Koca

**Affiliations:** Department of Urology, Medistate Kavacik Hospital, Istanbul, Turkey

To the Editor,

Rigicon^®^, Inc. (NY, USA) recently launched a novel corpus cavernosum dilator. The authors anticipate that the Rigicon^®^ HL Dilator^TM^, and any other purpose-designed new surgical tool introduced to surgeons’ use, has the potential to simplify the penile prosthesis implantation surgery. Consequently, in this article, we will demonstrate the features and the advantages and disadvantages of the HL Dilator^TM^ in the corpus cavernosum dilation technique.

The conventional penile prosthesis implantation surgery requires the dilation of each corpus cavernosum to facilitate cylinder/rod implantation. Historically, corporal dilation has been carried out with the use of Hegar dilators (originally designed for cervical procedures by German gynecologist Alfred Hegar) or Brooks dilators [1, 2]. Following corporotomy, space for the penile implant cylinders is created in the corpus cavernosum with the help of Hegar dilators as gradually increasing diameters of the Hegar dilators are distally and proximally inserted in the corpus [3].

When a penoscrotal incision is preferred, the flat and rod-like design of the Hegar dilator hinders particularly the distal dilation process as the surgeon’s hand is constantly blocked by the patient’s scrotum. In addition, even though the Brooks dilators are exempt from this limitation, the blunt tips of the wider diameter Hegar dilators limit proper dilation of the distal tips. When not appropriately dilated, the residual cavernous tissue may prevent proper measurement of the corpus cavernosum, leading up to the choice of a shorter than ideal size cylinder, preventing the penile implant cylinder tips from resting nicely underneath the glans penis, and consequently sometimes resulting in hypermobility of the glans (Concorde deformity) [4].

HL Dilators^TM^, which are already available and in use in the US, look promising in terms of the benefits it brings to the corporal dilation process.

The HL Dilator^TM^ are manufactured from medical grade stainless steel (316L) with an innovative design exclusive to corpus cavernosum dilation. HL Dilator^TM^ tips are designed to offer two different dilation diameters in one tool. The dilators are offered in 4 sizes: 9–10 mm; 11–12 mm; 13–14 mm; and 10–12 mm.

**Figure 1.**
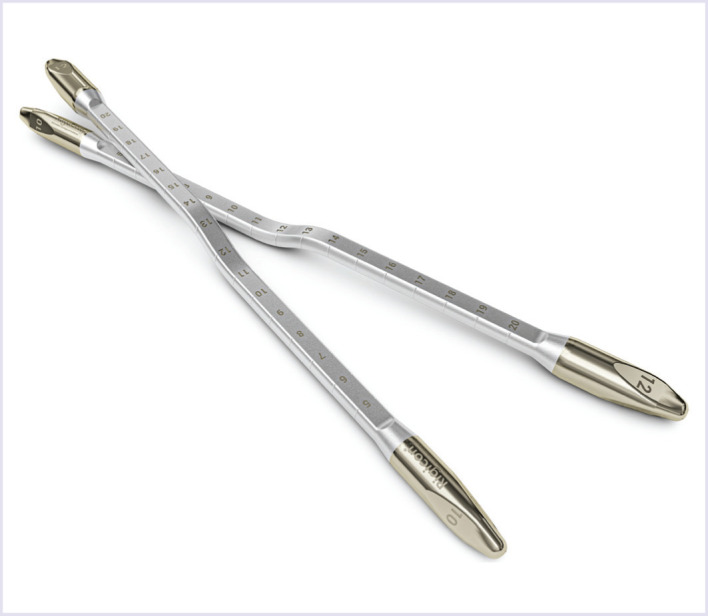
HL Dilator^TM^ with 10–12 mm tips.

The HL Dilator^TM^ with 10 mm tip on one end and 12 mm tip on the other end (Fig. 1) is a unique tool as this tool has the potential to be used as the single tool required for corporal dilation for an inflatable penile prosthesis implantation, rather than having to insert a series of dilators inside the corpus cavernosum.

HL Dilators^TM^ are lengthier compared to the Hegar dilators. This additional length is advantageous, especially when a subcoronal approach is preferred for penile prosthesis implantation. Due to the 25 cm length of the HL Dilators^TM^, even from a subcoronal corporotomy, the surgeon can dilate the corpus cavernosum down to the crus of penis in one single move and reliably measure the corpus. In most cases, the distance from the subcoronal corporotomy to the crus of penis is longer than the Hegar dilator’s length [5].

Furthermore, as the tips are not as thick and blunt as the Hegar dilators, the HL Dilator^TM^ enables proper dilation up to the tip of the glans penis and consequently minimizes the risk of Concorde defect. HL Dilator^TM^ tips, regardless of the dilator’s diameter, start from 6 mm and gradually widens to reach their final size. Thanks to this gradually increasing design of the dilator tips, dilation of the corporal body requires minimum force from the user. However, it leads to perforation with more force from the user. This is a disadvantage of HL Dilator^TM^.

One additional design advantage, the HL Dilator^TM^ has over the Hegar dilators, is the innovative shape of the dilator body. As in Brooks dilators, the S-shaped angle on the center of the tool creates a level difference between the two opposing tips (approximately 10 mm). This difference in level enables easier distal dilation by preventing the surgeon’s dilator handling hand from being blocked by the patient’s scrotum.

In addition to the points discussed above, all HL Dilators^TM^ also act as a precise sizer. When using a Hegar dilator, the surgeon must use a separate sizer tool, whereas, with the HL Dilator^TM^, the surgeon can size the corpus cavernosum with the same dilator as he or she completes the dilation process [6]. Because a single tool has two varying dilator tips, the authors expect a positive post-operative outcome regarding inflammation and infection since fewer surgical tools are introduced inside the corpus cavernosum.

**Figure 2.**
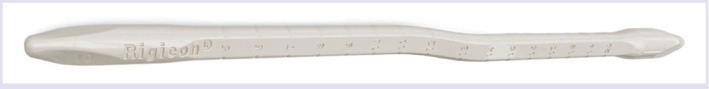
HL Dilators^TM^ – Single use model.

On a final note, aside from the reusable stainless-steel models, the HL Dilators^TM^ are also available manufactured from a medical-grade polymer and provided as sterile (Fig. 2). The benefit of the polymer HL Dilators^TM^ is that they do not require any reprocessing or sterilization before use. The surgeon has the option to use a new and sterile HL Dilator^TM^ for each case. This approach has the potential to minimize the risk of infection as improper cleaning and sterilization of the surgical tools may represent a source of infection for patients undergoing penile prosthesis implantation [7]. We assume that the sterile HL Dilators^TM^ will be an appropriate tool for mentor surgeons who frequently travel to perform penile prosthesis implantation workshops.

Our limited hands-on experience with the HL Dilators^TM^ demonstrates an easier, faster, and effective dilation process in comparison with Hegar dilators that we had used previously. The authors expect that following the increasing use of Rigicon^®^’s HL Dilators^TM^ at high volume centers worldwide, the experience generated in these centers may influence the approach, other implanting surgeons will have on this novel corporal dilation tool.
